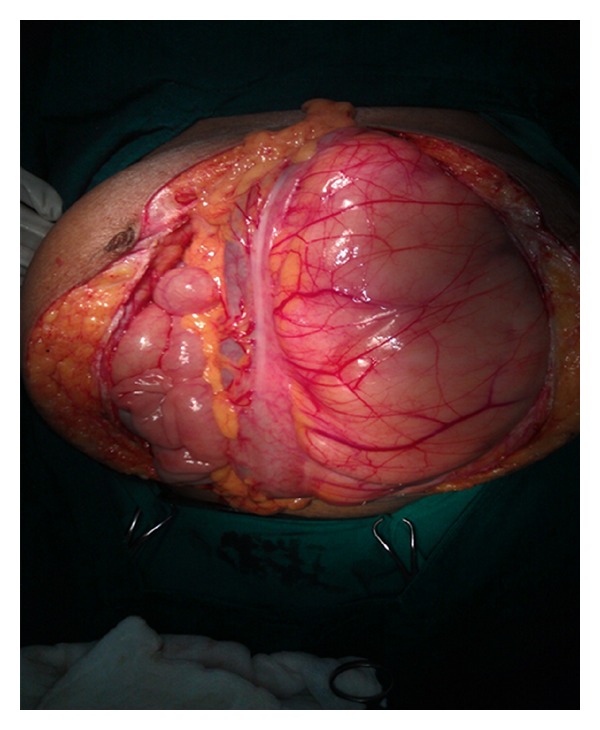# Giant Retroperitoneal Liposarcoma: A Case Report

**DOI:** 10.1155/2012/869409

**Published:** 2012-11-26

**Authors:** Chavan Shahaji, Parasnis Amit, Punia Prashant, Tekade Sachin

**Affiliations:** ^1^Department of Surgery, Padmashree Dr. D. Y. Patil Medical College Hospital & Research Centre, Pimpri, Pune 411018, India; ^2^Padmashree Dr. D. Y. Patil Medical College Hospital & Research Centre, Pimpri, Pune 411018, India; ^3^Department of General Surgery, Padmashree Dr. D. Y. Patil Medical College Hospital & Research Centre, Pimpri, Pune 411018, India

## Abstract

Soft tissue sarcomas represent less than 1% of all human neoplasms. One-third of malignant tumors that arise in the retroperitoneum are sarcomas and liposarcoma is the most common retroperitoneal sarcoma. More often than not, patients report late to the hospital due to the slow progress and few late symptoms. Thus, the tumor is known to grow to enormous sizes. Here, we report a case of giant retroperitoneal liposarcoma weighing more than 7 kgs.

## 1. Introduction 

Liposarcomas (Figure 1) generally present at diverse locations, such as upper and lower extremities, trunk, head and neck, retroperitoneum, and mediastinum. The peak incidence is in the age group of 50–70 years. Well-differentiated liposarcoma is the most common histological subtype encountered.

## 2. Case Report

A 65-year old lady presented with complaints of painless distension of abdomen for the last 2 years, which was insidious in onset and gradually progressive in nature. There was history of weight loss for the past 6 months. There was no history of hemoptysis, malena, and bleeding PR. Past History was not suggestive of any chronic illness.

 On examination she was: afebrile; pulse: 88/min and regular; blood pressure—136/80 mm Hg.

Her abdomen was distended; flanks were full, soft, and nontender. A palpable retroperitoneal lump of size approximately 26 × 18 cm occupying left hypochondrium, left lumbar, and left iliac fossa, involving umbilical region and crossing midline was felt with no evidence of ascitis. Rest of systemic examination was within normal limits and a provisional diagnosis of a retroperitoneal lump was made.

## 3. Investigations

Lab investigations were as follows: hemoglobin: 11.4 gm%; total leucocyte count: 4,600/cu mm of blood; polymorphs: 60%, lymphocytes: 30%; ESR: 18 mm/hour; random blood sugar: 88 mg/dl; liver and renal profile: WNL.

Chest X-Ray PA view was WNL and USG-guided fine needle aspiration cytology revealed it to be a lipoma.

Ultrasonography of abdomen was suggestive of “a large echogenic lesion having anechoic areas within it suggestive of necrosis, occupying the entire abdominal cavity, causing displacement of the adjacent bowel loops.” All other vital organs appeared normal and there was no evidence of ascites.

Contrast Enhanced Computed Tomography of abdomen revealed “a large heterogeneous lesion having multiple linear septation and HU value ranging from −97 to −105 HU suggestive of fat containing lesion causing compression and displacement of adjacent bowel loops, left kidney, and aorta towards the right side. This most likely suggests a malignant tumor most likely to be liposarcoma.”

## 4. Operative Findings

Mass was approached by a midline incision and descending colon was seen to be pushed across the midline entire left side of abdominal cavity was occupied by the mass. Extensions of the mass were seen in the entire abdominal cavity. Careful and complete resection of the mass was done and no organ resection was required. The resected out specimen weighed 7 kilograms.

Histopathological examination was indicative of a “well-differentiated liposarcoma.” 

Postoperative course was largely uneventful and the patient was discharged on the 5th postoperative day and is on regular followup since 2 years with no recurrence of tumor till date. Radiotherapy was not given considering the age of the patient and tumor extent.

## 5. Discussion

Liposarcoma is the most frequent histological type of retroperitoneal sarcoma, corresponding to 41% of these tumors [1]. Liposarcomas are subdivided into four well recognized subgroups based on morphology and cytogenic abnormalities: well differentiated, dedifferentiated, myxoid/round cell, and pleomorphic [2]. Liposarcoma occurs most commonly in the extremities (52%), retroperitoneum (19%), and inguinal region (12%) [3]. It has been reported that 20% of the tumors are >10 cm at the time of presentation [4]. These tumors of mesodermal origin are known to reach significant dimensions, despite their poor vascularization and can grow to enormous size, weighing over 100 pounds. In the literature, 18 and 42 kg liposarcomas have been reported [1]. These tumors usually present late with symptoms like vague abdominal discomfort, weight loss, and lump abdomen. Little precise information is available regarding effectiveness of various therapies and radical excision of tumour remains the treatment of choice. Excision may necessitate a continuous organ resection with kidney being the most commonly resected organ. The prognosis varies depending on the site of origin, the type of cancer cell, the tumor size, the depth, and proximity to lymph nodes. Well-differentiated liposarcomas and myxoid liposarcomas have a good prognosis and their rates of metastasis are low compared to the other types.

This case report is amongst the heaviest reported in the Indian subcontinent.

## Figures and Tables

**Figure 1:**